# Africa’s Population

**Published:** 1882-11

**Authors:** 


### AFRICA'S POPULATION.

From an address by Rev. Dr. R. S. Storrs, it appears that Africa contains a population of 200,000,000—more than twice the population of the Western Hemisphere. Its agricultural and mineralogical resources are inexhaustible. The interior of it is neither a sandy wilderness nor a series of marsh lands. The coast, which is noted everywhere for its malaria, presents no fair indication of the interior. Here is an almost unbroken succession of table lands rising everywhere from 2,000 feet to 2,500 feet high ; here are mountains larger than any in this country or in Europe ; a system of lakes surpassing even the magnificence of our own. Victoria Lake is larger in area than the whole State of New York ; mighty rivers flow through the country, and the climate is healthful and delightful. This is the country which commerce is bound to develop within the next fifty years. It has been said that Africa is like Noah's ark, which had few men but many beasts. The truth is that the human inhabitants are almost beyond count. There are races among them who are just as different from one another as the Turk from the Russian, and the Frenchman from the Chinaman. And many of them are highly susceptible to cultivation. Around this immense continent commerce has been hovering for many years. It is now on the point of making its way into it, and its progress will be attended by the grandest results. Just as great inventions burst upon the world and a dozen minds claim the first thought in the direction of their accomplishment, so the nations of the world seem to have turned their attention to this great "dark continent" as with one mind. England, Belgium, France, Italy and Russia have sent out scientific parties there and commercial embassies to increase our knowledge of the country. There are now steamship lines to the coast of Africa from Italy, France, England and the United-States. There are several steamship lines on the rivers of Africa. Railroad construction has been prosecuted vigorously. One road is to be built from the northern coast south through the desert of Sahara. This is the enterprise of an English company. There is already telegraphic communication from the Cape of Good Hope to England, and there will soon be connections from the former point to the northern coast of the continent. The country's wealth is almost boundless. There are gold and silver, diamonds from the South African mines, coal, iron, tin, copper, malachite, cotton and wool. One million pounds of coffee a year are exported from one district ; ostrich feathers, tobacco, hard woods and paper stock are other sources of wealth. Commerce is certain soon to possess this great continent of Africa.

